# Development of Extended-Release Mini-Tablets Containing Metoprolol Supported by Design of Experiments and Physiologically Based Biopharmaceutics Modeling

**DOI:** 10.3390/pharmaceutics14050892

**Published:** 2022-04-19

**Authors:** Michele Georges Issa, Natalia Vieira de Souza, Bruna Wenyi Chuang Jou, Marcelo Dutra Duque, Humberto Gomes Ferraz

**Affiliations:** 1 Department of Pharmacy, Faculty of Pharmaceutical Sciences, Universidade de São Paulo-USP, Av. Prof. Lineu Prestes 580, São Paulo 05508-080, SP, Brazil; nataliavieirasouza@hotmail.com (N.V.d.S.); brunawenyi@gmail.com (B.W.C.J.); sferraz@usp.br (H.G.F.); 2 Department of Pharmaceutical Sciences, Institute of Environmental, Chemical and Pharmaceutical Sciences, Universidade Federal de São Paulo—UNIFESP, Rua São Nicolau, 210 Centro, Diadema 09913-030, SP, Brazil; marcelo.duque@unifesp.br

**Keywords:** bioequivalence, DOE, GastroPlus^®^, metoprolol succinate, mini-tablets, PBBM, virtual bioequivalence study

## Abstract

The development of extended-release dosage forms with adequate drug release is a challenge for pharmaceutical companies, mainly when the drug presents high solubility, as in Biopharmaceutics Classification System (BCS) class I. This study aimed to develop extended-release mini-tablets containing metoprolol succinate (MS), while integrating design of experiments (DOE) and physiologically based biopharmaceutics modeling (PBBM), to predict its absorption and to run virtual bioequivalence (VBE) studies in both fasted and fed states. Core mini-tablet formulations (F1, F2, and F3) were prepared by direct compression and coated using nine coating formulations planned using DOE, while varying the percentages of the controlled-release and the pore-forming polymers. The coated mini-tablets were submitted to a dissolution test; additional formulations were prepared that were optimized by simulating the dissolution profiles, and the best one was submitted to VBE studies using GastroPlus^®^ software. An optimized formulation (FO) containing a mixture of immediate and extended-release mini-tablets showed to be bioequivalent to the reference drug product containing MS when running VBE studies in both fasted and fed states. The integration of DOE and PBBM showed to be an interesting approach in the development of extended-release mini-tablet formulation containing MS, and can be used to rationalize the development of dosage forms.

## 1. Introduction

The current challenge for pharmaceutical companies is to offer differentiated drug products that, in addition to unquestionable quality, are developed in a short period of time and with resource optimization. Within this scenario, the reformulation of commercially available drug products, combined with the use of statistical tools and physiologically based biopharmaceutics modeling (PBBM), has helped formulation scientists to develop medicines in a rational and effective way [1,2,3,4].

When it comes to controlled-release oral-dosage forms, the use of mini-tablets is a very interesting option, since they can be easily obtained and coated, and allow the possibility of modulating the release profile of the drug according to the mixture of different units [5,6].

Another factor that contributes to their use refers to the possibility of incorporating a large amount of drug (high drug load) in its units. Some studies report that the considerable decrease in the size of the punches can facilitate the direct compression of the drugs [7,8].

Metoprolol succinate (MS) is a selective β-1 receptor antagonist used to control hypertension and heart failure with reduced ejection fraction [9]. It is usually orally administered, being absorbed in the gastrointestinal tract and extensively metabolized in the liver, with a bioavailability of approximately 40% and an average half-life between 3 and 4 h in adults [10], which means that it is a drug that requires half-life (T_1/2_) modulation to reduce the quantity of daily doses.

In silico tools may be useful to rationalize this process, as PBBM encompasses many parameters, such as dissolution specifications, critical material attributes, food effects, and intra- and inter-subject variability, which may affect product performance, to predict in vivo drug release and absorption [1]. As such, since PBBM consists of an approach that links in vitro dissolution data and mechanistic oral-absorption modeling [11], it can contribute to rationalizing pharmaceutical product development by predicting in vivo performance [12] and establishing a safe space, which may be achieved by virtual bioequivalence (VBE) simulations [1]. In addition, it may be useful in an orally administered product’s biowaiver process [11].

GastroPlus^®^ is an in silico tool used to build physiologically based pharmacokinetic (PBPK) and compartmental models, which allows the linking of the dissolution profile obtained in vitro and the mechanistic oral-absorption modeling. This software was applied to simulate pharmacokinetic and absorption profiles of a large number of drugs, including reference and generic formulations of MS. However, although Basu et al. [13] performed simulations to compare the absorption profiles of generic and reference drug products containing MS, models and simulations of the fed state, essential for bioequivalence studies of extended-release formulations, were not constructed.

The present study aimed to develop metoprolol succinate extended-release mini-tablet formulations using design of experiments (DOE) and PBBM in fasted and fed states to predict the drug’s plasma-concentration profiles. The models were used to compare the best in-house formulation with its reference drug product through a virtual bioequivalence study.

## 2. Materials and Methods

### 2.1. Materials

To prepare the mini-tablets, the active pharmaceutical ingredient (API) metoprolol succinate (MS) was kindly provided by Libbs Farmacêutica Ltd.—São Paulo, Brazil, and the excipients used for formulations were: Microcel^®^ MC-102 microcrystalline cellulose (Blanver, Cotia, Brazil); colloidal silicon dioxide (Henrifarma, São Paulo, Brazil); titanium dioxide (Henrifarma, São Paulo, Brazil); magnesium stearate (Mallinckrodt^®^, Phillipsburg, NJ, USA); hypromellose (HPMC) Methocel^®^ K100M (Colorcon, Cotia, Brazil); Kollicoat^®^ IR Brilliant Blue (Basf, Ludwigshafen, Germany); Kollicoat^®^ IR White II (Basf, Ludwigshafen, Germany); Kollidon^®^ SR (Basf, Ludwigshafen, Germany); Kollicoat^®^ SR 30D (Basf, Ludwigshafen, Germany); and talc (Indukern do Brasil Química Ltd., Osasco, Brazil).

Aiming to evaluate the developed formulations, the analytical grade reagents monobasic potassium phosphate and sodium hydroxide (Casa Americana, São Paulo, Brazil) were used to prepare the dissolution medium. For sample analysis, monobasic potassium phosphate, orthophosphoric acid, and acetonitrile, all HPLC grade (Merck KGaA, Darmstad, Germany) were used to prepare the mobile phase.

### 2.2. Preparation of Mini-Tablets

Three MS mini-tablet formulations (F1, F2, and F3) were prepared as cores for coating through a direct-compression process (batch size of each formulation = 350 g), using a Fabbe eccentric compressing machine (Fabbe-Primar, São Paulo, Brazil) and multiple biconcave punches with a 3.0 mm diameter. The compositions of the formulations are described in Table 1.

Firstly, the components (Table 1) were weighed on a Shimadzu^®^ BL3200H analytical balance (Shimadzu^®^ Corporation, Kyoto, Japan). All of the powders, except the magnesium stearate, were sieved in a 1.18 mm mesh (Bronzinox, São Paulo, Brazil). They were transferred in sequence to a polyethylene bag and manually mixed for 10 min. After this step, the magnesium stearate was sieved into a 0.42 mm mesh (Bronzinox, São Paulo, Brazil) and added to the other components for final mixing for 2 min. Finally, the material was transferred to the tablet press machine.

#### 2.2.1. Characterization of Mini-Tablets

The core mini-tablets (F1, F2, and F3) were characterized in terms of average weight, hardness, friability, true density, and drug content.

Twenty units of each formulation were used to determine the average weight, with each one being individually weighed on a Shimadzu^®^ BL3200H analytical balance (Shimadzu^®^ Corporation, Kyoto, Japan).

The hardness was evaluated in a Logan^®^ HDT-300 Hardness Tester (Logan Instruments Corp., Somerset, KY, USA), using 10 units of each formulation.

For the friability test, a procedure similar to that standardized by Issa et al. [14] was used, but adapted for mini-tablets: about 5 g of each formulation was weighed on a Shimadzu^®^ BL3200H analytical balance (Shimadzu^®^ Corporation, Kyoto, Japan) and transferred to the Logan^®^ FAB-2 friabilometer (Logan Instruments Corp., Somerset, KY, USA), along with 100 glass spheres that were 4.3 mm in diameter. The test was carried out until reaching 200 rotations, after which the mini-tablets were collected from the equipment and subjected to a new weighing to determine the mass after abrasion. The friability was calculated using Equation (1):

(1) $$\mathrm{Friability}(\%)=\frac{\left(Mf-Mi\right)}{Mi}\times 100$$

where *M_f_* is the mass after abrasion and *M_i_* is the mass before abrasion.

The true density test was performed using a Helium Ultrapycnometer 1000 gas pycnometer (Quantachrome Corporation, Boyton Beach, FL, USA), in which about 4.0 g of each sample was subjected to five measurements of volume and density. The average density was calculated at the end of each test.

The MS content of each formulation was determined using approximately 120 mg of mini-tablets, which were ground in a porcelain mortar, and 100 mg of the powder was weighed on a Shimadzu^®^ BL3200H analytical balance (Shimadzu^®^ Corporation, Kyoto, Japan). The weighed material was transferred to a 100 mL volumetric flask containing approximately 60 mL of phosphate buffer at pH 6.8, which was left for 30 min in a Unique Cleaner USC2800A ultrasonic bath (Unique Group, Indaiatuba, Brazil). After this time, the volume of the flask was completed, and the solution was homogenized and filtered through a Sartorius Minisart RC 25 regenerated cellulose membrane (Sartorius AG, Goettingen, Germany) with a pore size of 0.2 µm. The absorbance of the resulting solution was evaluated in a Beckman Coulter—DU640 spectrophotometer (Beckman Coulter Inc., Fullerton, CA, USA), using a quartz cuvette with an optical path of 0.5 cm at a wavelength of 272 nm. The procedure was performed in triplicate for each formulation.

#### 2.2.2. Coating of Mini-Tablets

The core mini-tablets were coated using Mycrolab Hüttlin fluid bed equipment (Hüttlin GmbH, Steinen, Germany). To select the best core, as well as the adequate composition of the coating film, a 3^3−1^ fractional factorial design was carried out, using as variables and levels the core type (formulation F1, F2, or F3), coating weight gain (5%, 7.5%, or 10%), and percentage of pore-forming polymer (Kollicoat^®^ IR0, 4%, or 8%) in the coating film. The assay matrix is presented in Table 2. For each coating assay, 50 g of core mini-tablets was used. All parameters used in this process are described in the Supplementary Materials (Table S1).

The coating film was formed by combining Kollicoat^®^ SR 30D as the controlled-release polymer and a 50:50 *w*/*w* mixture of Kollicoat^®^ IR White II and Kollicoat^®^ Brilliant Blue as the pore-forming polymer, in the amounts indicated in the DOE (Table 2). In all cases, the amounts of talc and titanium dioxide were adjusted to complete 5% of pigments. The compositions of the coating films are shown in the Supplementary Materials (Table S2).

### 2.3. In Vitro Studies

#### 2.3.1. Dissolution Method

The dissolution protocol used for the coated mini-tablets was adapted from the method recommended by the FDA [15] for metoprolol succinate/hydrochlorothiazide association, which uses Selopress Zok^®^ (Metoprolol succinate 95 mg/hydrochlorothiazide 12.5 mg—AstraZeneca^®^) as the reference product.

To evaluate the developed formulations, capsules no. 1, containing 16 mini-tablets (equivalent to 95 mg of MS) each, were placed into sinkers (anchors). The dissolution tests were performed using 900 mL of potassium phosphate buffer at pH 6.8 as the dissolution medium, at a temperature of 37 °C and a rotation speed of 100 rpm, using a paddle and sinker, with 5 mL aliquots collected with a VK 8000 Automatic Dissolution Sampling Station (Agilent Technologies Inc., Santa Clara, CA, USA) and filtered with a polyethylene membrane (Quality Lab Accessories L.L.C., Bridgewater, NJ, USA) with a 45 µm pore size at the following times: 0.5, 1, 2, 4, 6, 8, 10, 12, 16, 20, and 24 h.

The filtered samples were quantified as described in Section 2.3.2. The dissolution test of the reference product was carried out using the same test conditions. Three units of each formulation (n = 3) and twelve units (n = 12) of the reference drug product were evaluated.

#### 2.3.2. Drug Quantification in the Dissolution Test

High-performance liquid chromatography (HPLC) equipment (HITACHI ELITE LaChrome L-2000, VWR Hitachi, Tokyo, Japan), a HITACHI ELITE LaChrome L-2130 pump (VWR Hitachi, Tokyo, Japan), a HITACHI ELITE LaChrome Diode array L-2455 detector (VWR Hitachi, Tokyo, Japan), and the EZChrom Elite program for data processing were used.

A LiChroCart C_18_ column (250 mm × 4.6 mm × 5 μm—Merck KGaA, Darmstad, Germany) was the stationary phase, while the mobile phase was 80% 0.05 M phosphate buffer at pH 3.0 and 20% acetonitrile with a flow of 1.2 mL/min. The injection volume was 20 μL, with detection performed at 222 nm at a monitored temperature of 25 °C [16]. To insert samples in vials, a Sartorius Minisart RC 25 (Sartorius AG, Goettingen, Germany), a regenerated cellulose membrane syringe filter with a 0.45 µm pore size, was used, while always discarding the previously used 2 mL of each solution for membrane saturation. Using the quantification standard, a metoprolol succinate analytical curve was obtained while adopting the same conditions used for the samples.

### 2.4. Statistical Analysis of the Design of Experiments

To assess which factors would have an influence on the release of MS from the coated mini-tablets, a statistical analysis of the DOE was performed using the Statistica 11.0 program (StatSoft Inc., Tulsa, OK, USA). The dissolution efficiency (DE%) was evaluated as a dependent variable, and was calculated using the Microsoft Excel add-in DDSolver [17].

### 2.5. Additional Formulations

Based on the analysis of the experimental design, three more coated formulations (E10, E11, and E12) were prepared and compared to the reference drug product. For this purpose, core mini-tablets of F1 were used, as well a 10% coating weight gain and the pore-forming polymer Kollicoat^®^ IR (1% E10, 2% E11, and 3% E12). The compositions of the coating films are described in the Supplementary Materials (Table S2).

The amount of MS dissolved was analyzed, and complementary to this, the DE% was calculated, as well as the similarity factor (*f*2) between the reference drug product and the formulation with the most promising results. DE% and *f*2 were calculated using the Microsoft Excel add-in DDSolver [17].

The dissolution tests of these formulations and the quantification of the amount of the dissolved drug were carried out as described in Section 2.3.1 and Section 2.3.2.

### 2.6. Formulation Optimization

With the objective of optimizing the formulations, the dissolution profiles of ternary mixtures of mini-tablets of the formulations F1, E11, and E12, which were previously described, were simulated. To list the number of combinations, an experimental mixture design (Table 3) was planned using the computer program Design Expert, version 9, 2014 (Stat-Ease, Inc. Minneapolis, MN, USA). The first component of the mixture (F1) was kept fixed, and combinations were carried out for a total of 16 mini-tablets (equivalent to 95 mg of metoprolol succinate) according to the restrictions imposed for the other formulations: E11 (1 to 5 mini-tablets) and E12 (10 to 15 mini-tablets).

To simulate the dissolution profiles of the mixtures, a spreadsheet was built using Microsoft Excel (2013 version) from the dissolution profiles of formulations F1, E11, and E12. To calculate the percentages dissolved at different time points, Equation (2) was used:

(2) $$Q\%Mtc=\left(\left(\left(\frac{Q\%F1tc}{n}\right)\times nF1\right)+\left(\left(\frac{Q\%E11tc}{n}\right)\times nE11\right)+\left(\left(\frac{Q\%E12tc}{n}\right)\times nE12\right)\right)$$

where:

*Q%Mtc*: percentage dissolved of the mixture at its collection time (*tc*);
*Q%F*1*tc*: percentage dissolved of formulation *F*1 at time *tc*;
*Q%E*11*tc*: percentage dissolved of *E*11 formulation at time *tc*;
*Q%E*12*tc*: percentage dissolved of *E*12 formulation at time *tc*;
*n*: total amount of mini-tablets;
*nF*1: number of mini-tablets of the formulation *F*1;
*nE*11: number of mini-tablets of the formulation *E*11;
*nE*12: number of mini-tablets of the formulation *E*12.

The optimized formulation was defined based on the statistical analysis of the simulated dissolution profiles of the mixtures of mini-tablets, considering the percentages of MS dissolved at the following time points as responses: 2 h (Q%_2h_); 4 h (Q%_4h_); 10 h (Q%_10h_), and 20 h (Q%_20h_).

Guided by the results, an optimized mixture (FO) was prepared. The dissolution profile of FO (n = 12) was evaluated using the reference drug product (n = 12) as a comparison, and using the previously described dissolution and quantification methods. Additionally, zero-order, Higuchi, and Korsmeyer–Peppas kinetic models were applied using DDSolver [17].

### 2.7. Development, Verification, and Application of a Pharmacokinetic Model for Metoprolol

**Figure 1:**
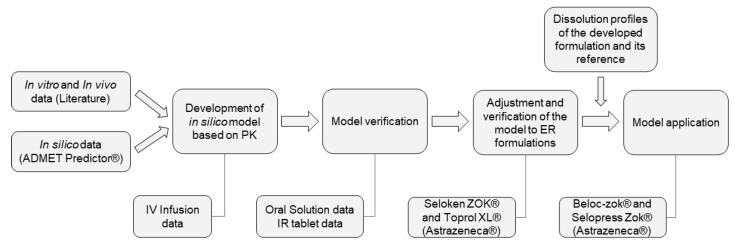
Major steps of the PBBM approach.

A compartmental pharmacokinetic model for metoprolol was developed, verified, and applied to the experimental dissolution data using a physiologically based biopharmaceutics modeling (PBBM) approach (Figure 1).

#### 2.7.1. Development and Verification of the Pharmacokinetic Model

The PKPlus™ module in GastroPlus^®^ version 9.8 (Simulations Plus Inc., Lancaster, CA, USA) was used to develop the pharmacokinetics (PK) model. For this purpose, a plasma-concentration profile of 5 mg IV infusion of metoprolol obtained from the literature [18] was used to build the compartmental PK model. Thereby, data obtained from the literature or obtained from the chemical structure of the drug, using the ADMET Predictor^®^ module in GastroPlus^®^, as summarized in Table 4, in addition to the 5 mg IV data, were used to set the model.

A simulation was run while considering IV:Infusion as the dosage form: 5 mg for the dose and 10 min for the infusion time. The predicted plasma concentration-time curve was compared with the observed one [18].

The model was verified in sequence by applying it to a 10 mg IV infusion while considering the infusion time of 5 min and comparing the predicted plasma curve with the observed data [18].

#### 2.7.2. Model Verification and Adjustments to ER Formulations

After verification of the model using IV data, it was used to simulate oral-dosage forms. When using the gut physiology default settings (Human-physiological-Fasted; Opt logD Model SA/V 6.1), it was necessary to set the dosage form to IR:Solution and IR:Tablet, respectively, to predict the absorption of 50 mg of oral solution (OS), as well as a 50 mg immediate release (IR) tablet. The predicted plasma concentration-time curves were compared to the literature data [13,23].

Then, the developed PK model, in a fasted state, had its absorption scaling factors (ASFs) adjusted for extended-release (ER) tablets. This was necessary due to the controlled release of this dosage form, which is modulated by the composition of the pellets in the reference product, and happens over time. Thereby, the selected dosage form was CR:Dispersed, which is adequate for controlled-release pellets. Using the Optimization module in GastroPlus^®^, and the plasma concentration-time curve of 25 mg Seloken ZOK^®^ (Astrazeneca^®^) from the literature [24], the ASFs were optimized for the absorption of ER formulations and, for verification, the same model was applied to a 200 mg Toprol-XL^®^ plasma concentration-time curve [25], an extended-release formulation also produced by Astrazeneca^®^, but nevertheless registered as reference drug product in different countries.

Moreover, the dissolution profile of Selopress ZOK^®^ (Astrazeneca^®^), 95 mg metoprolol succinate, was used as the model input, and the predicted curve was compared with data on Beloc-ZOK^®^, 95 mg metoprolol succinate, the same product also produced by Astrazeneca^®^, but registered under different names and commercialized in different countries, as described in the literature [26], aiming to show the predictability of the PK model.

Using the fasted model as a basis, the gut physiology was set to the fed-state-model default settings (Human-Physiological-Fed; Opt logD Model SA/V 6.1). Since metoprolol is a BCS class I drug, the plasma concentration of IR formulations was not available in the fed condition in the literature. Hence, the model ASFs were calibrated directly with ER formulations, starting with a 200 mg Toprol-XL (Astrazeneca^®^) plasma concentration-time curve [25] and then applying to another 200 mg profile obtained in the same study [25] for verification of the fed-state model.

#### 2.7.3. Evaluation of the Predictability of the Model

The predictability of the PBBM was evaluated in sequence using the percentage prediction error (%PE) and success criteria (SC). The %PE (Equation (3)) shows the variation between predicted and observed values. It should not exceed 15% to dispense with external validation [27], and it was used when the coefficients of variation (%CV) of C_max_ and AUC of the observed data were not available. SC (Equation (4)) gives a lower limit (LL) and an upper limit (UL) to the parameters, which are calculated considering the number of subjects (*n*) and %CV (Equation (5)), as described by Abduljalil et al. [28]. The %PE was applied to the PK data predicted for the IV, OS, and IR formulations, while SC was used for the ER formulations, in fasted and fed conditions.

(3) $$\%PE=100\times \left[\frac{Predicted\;Value-Observed\;Value}{Observed\;Value}\right]$$

(4) $$SC=exp\;\left[ln\left(x\right)\pm 1.96\;\frac{\sigma }{\sqrt{n}}\right]$$

(5) $$\sigma =\sqrt{ln[(\frac{CV\%}{100}})x^{2}+1]$$

where:

$\underline{x}$ = average of the parameter;
n = number of subjects.

#### 2.7.4. Application of the PK Model to the Developed Formulation

Finally, the PK model was applied to the optimized formulation (FO), which contained 95 mg of metoprolol succinate as mini-tablets; its dissolution profile was determined; and the single Weibull function was applied.

### 2.8. Virtual Bioequivalence Study

Virtual bioequivalence (VBE) studies were run in GastroPlus^®^ using the Population Simulator. The dissolution profiles of the optimized formulation (FO) and reference product (Selopress Zok^®^) were used as inputs for the VBE studies after applying the single Weibull function, as described earlier.

Since ER formulations must show bioequivalence in fasted and fed states, the VBE study was run in both conditions while adopting 90% confidence intervals (CI) for the AUC and the C_max_. The VBE studies were performed while considering 48 healthy subjects using a length of 48 h and 300 output data points.

## 3. Results and Discussion

The direct-compression method proved to be viable for the production of the immediate-release formulation (F1), as well as the controlled-release matrices (F2 and F3). Furthermore, the 40% concentration of the drug was very useful in reducing the number of units needed for the composition of the dose in each pharmaceutical dosage form.

The variation presented for the average weight of the mini-tablets (Table S3) was adequate, and obtained coefficients of variation (CV%) of 3.6%, 4.3%, and 4.7% for F1, F2, and F3, respectively. According to Lopes et al. [29], a CV% lower than 5% for mini-tablet formulations can be an indication of the weight homogeneity of the batches produced [30].

The true density value (Table S3) of the formulation that contained a greater amount of microcrystalline cellulose (F1) was higher than that of the others (F2 and F3), in which a large part of this component was replaced by the Kollidon^®^ SR and Methocel^®^ K100M polymers. Considering that this property was determined by the volume of the solid while excluding the volume occupied by the internal pores and spaces between the units, it appeared that in this case, as the process used was direct compression, the results may have been related to the density of each of the materials that were part of the formulation [31]. Taking as an example the true density values of microcrystalline cellulose (1.512–1.668 g cm^−3^) and HPMC (1.326 g cm^−3^), it was possible to observe that there was a difference in the density of these excipients [32], and that the difference observed may have been related to the amount of microcrystalline cellulose in the F1 formulation.

For materials that exhibit viscoelastic behavior, such as microcrystalline cellulose and polymers, especially hypromellose, the compression speed can influence the hardness of the tablets. According to the degree of viscosity of the polymer, the time of plastic deformation must be longer in order to fill the voids and form stronger bonds, resulting in a great rigidity of the core [33]. For the obtained formulations, F2 presented the highest hardness value (Table S3), which may have been related to the lower viscosity grade of Kolidon^®^ SR when compared to Methocel^®^ K100M [32,34]. A similar result was obtained by Lopes et al. [29] in the production of ibuprofen matrix mini-tablets, in which the increase in hardness was accompanied by a decrease in the viscosity of the HPMC.

On the other hand, the high hardness value (F2) did not result in lower friability; however, the results obtained for the three formulations were satisfactory, and were below 1% [30]. Regarding the drug content, the amount of MS present in the mini-tablets was close to the theoretical value of 40% (Table S3).

**Figure 2:**
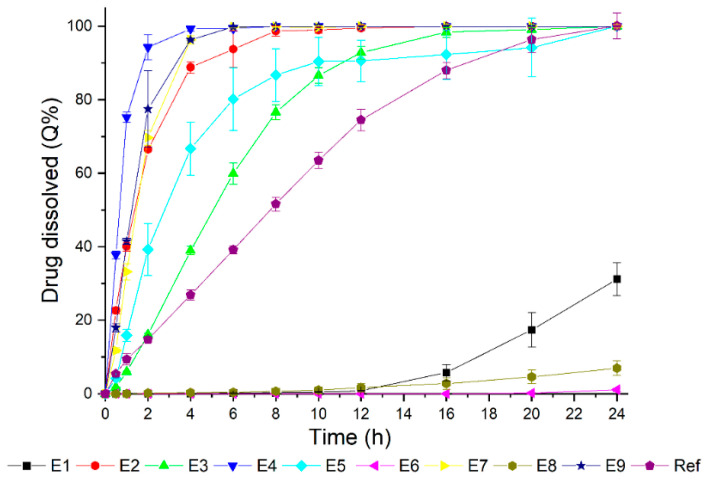
Dissolution profiles of the coated mini-tablet and reference product (Selopress Zok^®^) formulations, as evaluated in the following conditions: 900 mL of pH 6.8 phosphate buffer, paddle + sinker, and a 100 rpm rotation speed.

The dissolution profiles of all nine coated mini-tablet formulations (E1 to E9) are presented in Figure 2. The obtained dissolution profiles were divided into two groups in comparison to the reference drug product: the first group (E2, E3, E4, E5, E7, and E9) showed a high drug release, while the second one (E1, E6, and E8) showed a lower performance. This configuration indicated that an absence of the pore-forming polymer (Kollicoat^®^ IR) in the coating film’s composition (Table 5) compromised the drug release. Moreover, the matrix type did not suggest notable influences on the results, which were due to the combination of Kollicoat^®^ SR 30D and Kollicoat^®^ IR polymers.

However, to support these hypotheses, the results were statistically evaluated using the dissolution efficiency as the dependent variable (Table 5). Through the analysis of variance of the DE% (Table S4), the presence of the Kollicoat^®^ IR pore former proved to be the only factor with a significant influence on the evaluated response. Furthermore, when observing the F values, the importance of this component was much higher in comparison with the other factors.

**Figure 3:**
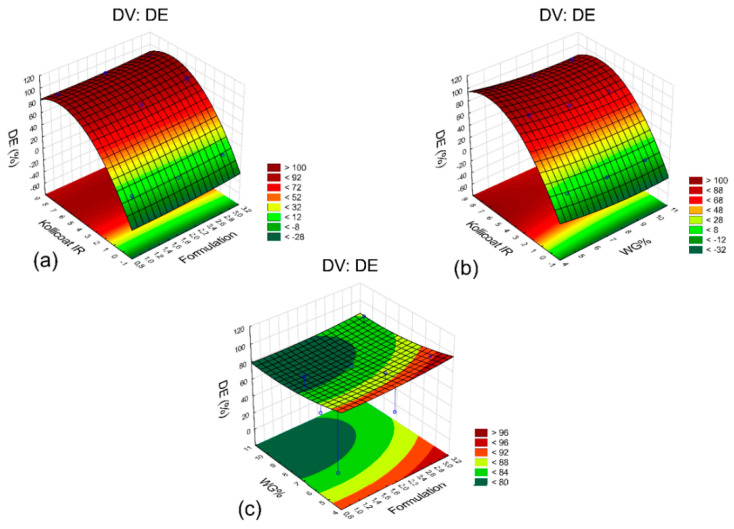
Response surface graphs for DE%, a dependent variable: (**a**) Kollicoat^®^ IR versus formulation; (**b**) Kollicoat^®^ IR versus WG%; (**c**) WG% versus formulation.

The response surface graphs allowed an easy visualization of the relation between DE% and the amount of Kollicoat^®^ IR (Figure 3a,b), regardless of the coating weight gain (WG%) and the type of core formulation used. This is indicated by the parallelism of the colors in the figure, which change according to the variation in the level of the pore former. A comparative analysis of the WG% and formulation variables (Figure 3c) indicated the interaction of these parameters, as the colored lines showed curvature, which made the interpretation of the results impracticable [35]. Nevertheless, it was observed that for lower levels of weight gain, as well as for higher level formulations, such as for F3 (HPMC K100M core), higher values of DE% could be obtained, indicating a possible relationship between these factors and the release of MS.

**Figure 4:**
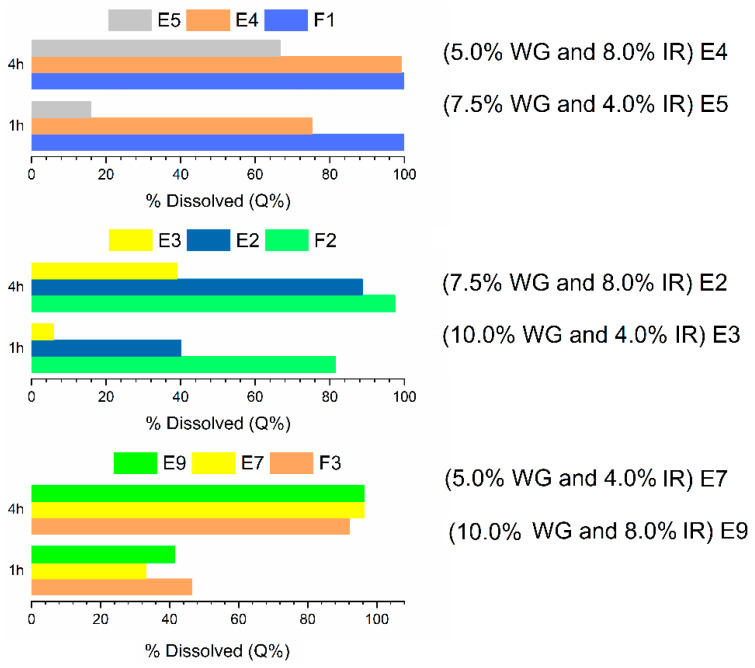
Comparison of Q%_1h_ and Q%_4h_ values of formulations F1, F2, and F3 before and after coatings. WG% and IR represent coating weight gain and Kollicoat^®^ IR, respectively.

To evaluate the influence of the core formulations of the mini-tablets on the dissolution performances, the results obtained with the initial formulations (F1, F2, and F3), before and after the coatings, were compared (Figure 4) while considering the time points Q%_1h_ and Q%_4h_.

Formulations E1, E6, and E8 were not considered in the comparative analysis, since the absence of the pore former resulted in a low MS release, as the Kollicoat^®^ SR30D was the only release agent. In a similar study, carried out by Mohamed et al. [6], an absence of theophylline release from the mini-tablet matrices was observed when the pore former was not added to the coating film, and this result was attributed to the low permeability of the controlled-release polymer.

In Figure 4, it was verified that the cores constituted by the formulations F1 (immediate-release) and F2 (Kollidon^®^ SR) presented a release modulated by the combination between the thickness of the coating layer (WG%) and the amount of pore former (Kollicoat^®^ IR), as usually occurs in reservoir systems [6]. Coincidentally, from formulation E4 to E5, as well as from E2 to E3, there was an increase in the percentage of the controlled-release polymer and a decrease in the pore former. These factors led to a decrease in the percentage of drug dissolved, as observed through the reduction in the Q%_1h_ and Q%_4h_ values. Moreover, a 10% weight gain, applied in the E3 formulation, showed better efficiency in controlling the drug release.

However, it was not possible to modulate the dissolution of the MS for the formulations derived from the F3 core, which contained HPMC K100M. This was due to the combination of the pore-forming polymer in the coating film and the hydrophilic characteristic of the HPMC. As the dissolution of Kollicoat^®^ IR began, channels opened up in the coating film, and as the dissolution medium penetrated the mini-tablet, the HPMC began to gel, causing an internal pressure that ruptured the reservoir system [6,36]. From this moment, the formulations began to behave similarly to F3, with a high release of MS in the first hours of dissolution testing.

**Figure 5:**
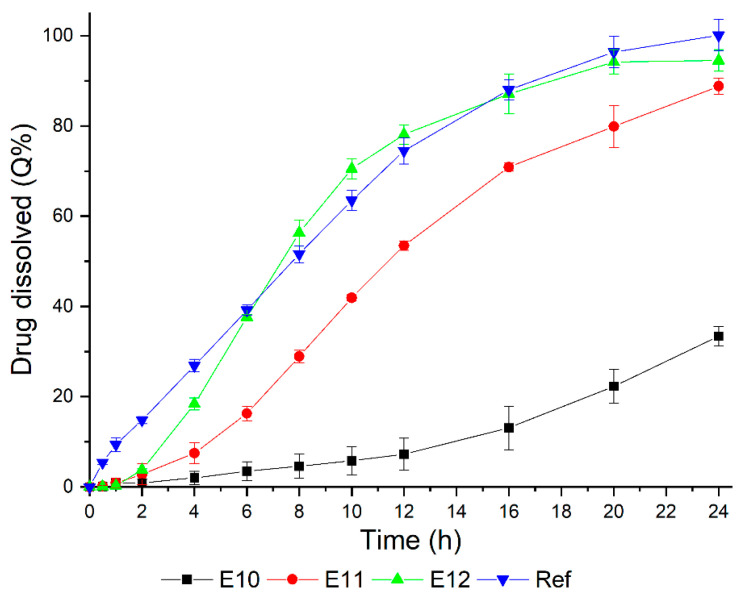
Coated mini-tablet formulations obtained in the optimization step and reference drug product (Selopress Zok^®^) dissolution profiles, evaluated using 900 mL of phosphate buffer at pH 6.8, paddle + sinker, and a 100 rpm rotation speed.

Following this, additional formulations were produced by adjusting the amount of Kollicoat^®^ IR to levels below 4% (E10, E11, and E12). In all cases, the F1 core was used without a polymer in the matrix and 10% weight gain. The dissolution results are shown in Figure 5 and Table 6.

Formulation E12, which contained 3% of the pore-forming polymer in the coating composition, presented the highest similarity with the dissolution profile of the reference drug product (Figure 5). Although Q%_1h_, Q%_8h_, and Q%_20h_ met the USP specification [37] and the DE% was close to the reference product’s values (Table 6), the release of the MS was low in the first four hours of the assay. So, for comparison purposes, the *f*2 value (58.4) was obtained. Then, this data indicated similarity between E12 and the reference product [38].

Dose fragmentation into small units allows multiparticulate systems to combine different release mechanisms in a single pharmaceutical dosage form. Thereby, it is possible to modulate the dissolution profile of a drug to achieve better therapeutic results [5,8,39].

Using this concept, the process was idealized to fine-tune the dissolution profile of the MS mini-tablets presented by the E12 formulation through the combination of formulations that had already been produced, and more precisely by creating different mixtures. For this optimization step, formulations E1 (uncoated), E11 (Kollicoat^®^ IR 2%/WG% 10%), and E12 (Kollicoat^®^ IR 3%/WG% 10%) were used.

The combinations followed the statistical mixture design, with the amount of formulation F1 kept constant (one unit). This was included due to the necessity of the improving drug release in the first hours of the dissolution testing. However, higher percentages were not tested, since any increase in the amount of MS dissolved at the beginning of the test would cause a relevant impact on the percentage of drug dissolved in the rest of the dissolution profile.

**Figure 6:**
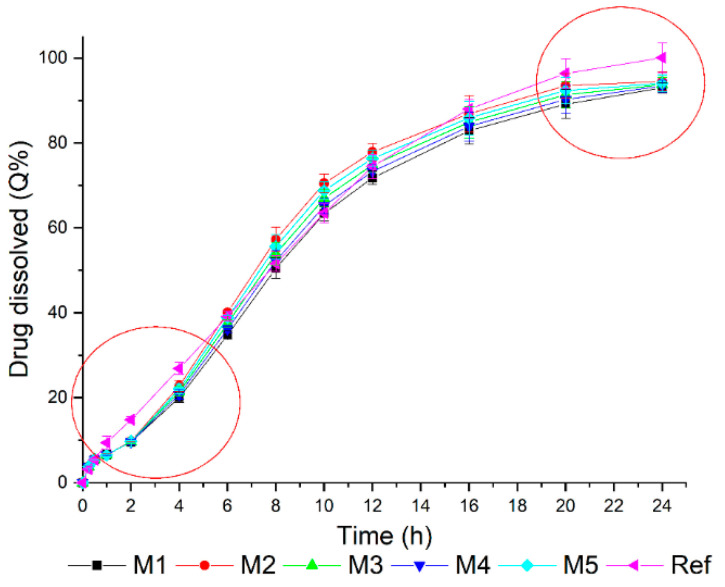
Simulated dissolution profiles for the mixtures of F1, E11, and E12 mini-tablets using Equation (2).

The dissolution profiles were simulated using Equation (2) (M1, M2, M3, M4, and M5), and the results are shown in Figure 6. The obtained dissolution profiles were highly similar, and—as highlighted in the graph—the optimization should be based between the points of 2 and 4 h and above 20 h. Considering these results, the percentages of drug dissolved in these points, as well as an intermediate time point (10 h), were used as responses in the statistical evaluation of the optimized formulation (Table 7).

Each response was analyzed using the most appropriate mathematical model through multiple regression and ANOVA results. Table S5 shows the models used, as well as the respective values of p, R^2^, and R^2^_ajdusted_. *p*-values lower than 0.05 and high determination coefficients indicated the feasibility of the models in the interpretation of the obtained data [40].

The equations of the regression models are listed in Table S6, and all coefficients were considered significant within a 95% confidence interval.

Based on these data, it was possible to proceed with the optimization of the mixtures using the desirability function. Supported by the equations (Table S6), desirability was a tool that allowed the combination of all responses in a single measure, thus facilitating the prediction of adequate values for the independent variables according to the intended purpose [41]. The results obtained in the dissolution of the reference drug product, as well as those shown in Figure 6, were used in the selection of the optimization criteria (Table 8).

**Figure 7:**
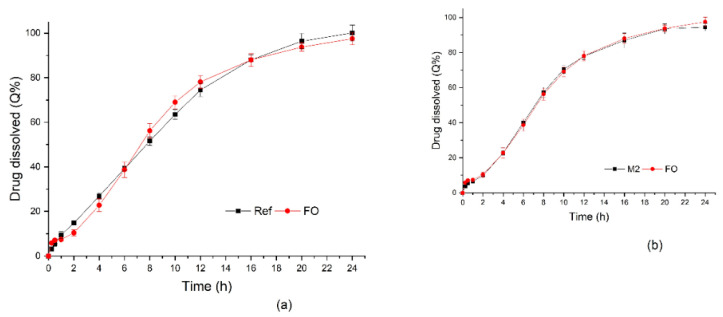
Dissolution profiles of (**a**) FO and Selopress Zok^®^ and (**b**) a comparison between simulated (M2) and real (FO) dissolution profiles. For FO and reference drug product, the tests were carried out in 900 mL of phosphate buffer at pH 6.8, using a paddle + sinker at a 100 rpm rotation speed.

The desirability value obtained was 0.972—close to 1, which meant that the suggested mixture (optimized formulation—FO) was able to meet the request made in the optimization [41]. The suggested mixture was: 1 F1 + 1 E11 + 14 E12. FO, in comparison with the simulated mixtures, and was revealed to have the same composition as M2. Although the dissolution profile of M2 presented differences with the reference product, the formulation proposed (FO) was tested while considering that the profiles of the original formulations (on which the simulation was based) were obtained by the average of only three determinations. To test this combination, 12 capsules of FO were prepared, and a dissolution test was performed. Then, the obtained dissolution profile was compared to that of the reference drug product (Figure 7a).

To validate the simulation predictability, the M2 and FO results were plotted (Figure 7b) to facilitate the comparison. This indicated the viability of the strategy adopted.

In addition to the previous comparison, the dissolution kinetics were evaluated applying three dependent models (zero-order, Higuchi, and Korsmeyer–Peppas) and the calculated similarity factor *f*2, the results for which are shown in Table 9. These kinetics-dependent models were selected to be tested due to the type of formulation (extended-release) that we developed in this study. If drug release occurred slowly, with a constant amount released per unit of time, the dissolution profile was fit to zero-order kinetics. Drug release can be related to the diffusion process, and is dependent on the square root of the time, as in the Higuchi model. Since the capsules contained mini-tablets with different release mechanisms, the dissolution profile could follow Korsmeyer–Peppas kinetics [38].

To select the model that best explained the drug-dissolution kinetics, the adjusted coefficient of determination (R^2^_adj_.), the Akaike criterion (AIC), and model selection criteria (MSC) were analyzed.

Table 9 shows that for both Selopress Zok^®^ and FO, the model that best fit the dissolution profiles was the Korsmeyer–Peppas model, since it presented the highest R^2^_adj_. and MSC, as well as the lowest AIC [16,42].

Since the reference drug product consisted of a multiparticulate system formed by small pellets and the optimized formulation FO was composed of mini-tablets, the release exponent (n) of the Korsmeyer–Peppas equation was calculated and used for data interpretation.

For n values between 0.43 and 0.85, the drug release is considered to be anomalous transport. In this case, the drug release was not only due to diffusion, but also occurred in a reservoir system [30,40]. Thus, it indicated that the coating film was not solely responsible for modulating the dissolution of the MS reference product. The results for the FO confirmed the composition of the mixture of mini-tablets, since among them there was an uncoated tablet responsible for the release of MS in the first hour of the dissolution test.

Finally, the *f*2 value obtained was 73.6. Considering that this was within the range of 50 to 100 [38], it converged with the kinetics results, showing the similarity between the dissolution profiles of Selopress Zok^®^ and the optimized FO formulation.

Using all these results, it was possible to carry out the VBE study. This part had as a first step the construction and selection of the pharmacokinetic model, which was fundamental to simulating the drug absorption.

The two-compartment pharmacokinetic model, which was obtained using the PKPlus™ module in GastroPlus^®^, was the most appropriate to fit the plasmatic concentration profile of 5 mg IV metoprolol [18], which showed an adequate coefficient of determination (R^2^ = 0.996). The PK parameters generated by the model are shown in Table 10.

The obtained elimination half-life, clearance, and volume of distribution values (Table 10) for the PK model were close to the values described in the literature: T_1/2_ = 3.2 ± 0.2 h [43]; CL = 67.5 ± 13.5 L/h, considering average weight of 75 kg [43]; and Vd = 2.2 ± 0.3 L/Kg [18].

**Figure 8:**
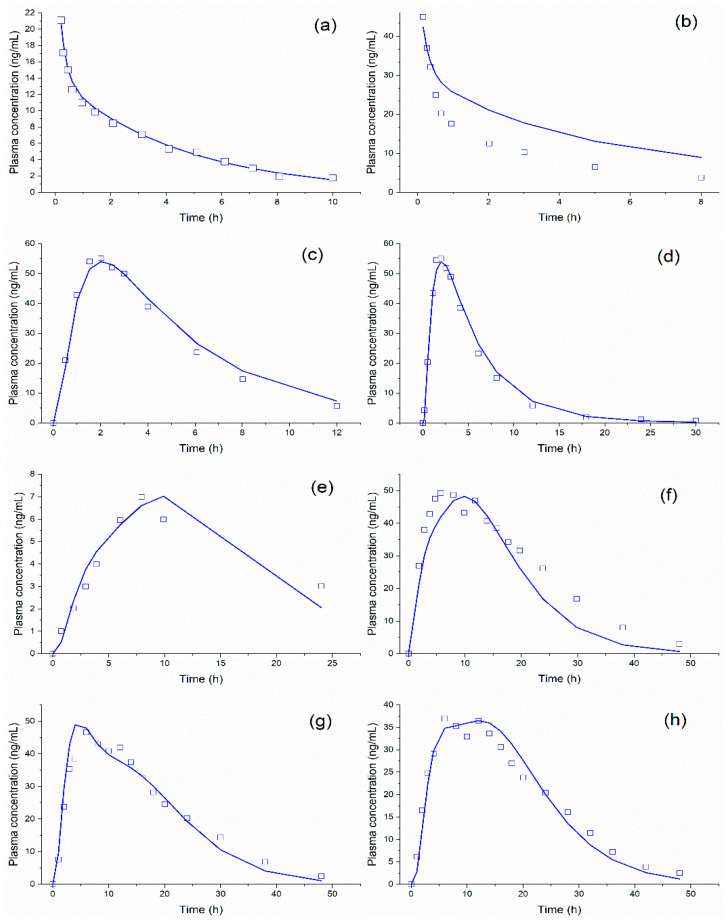
Observed (squares) and simulated (lines) plasma curves of metoprolol while considering the fasted-state 5 mg IV infusion (**a**), 10 mg IV infusion (**b**), 50 mg IR tablet (**c**), 50 mg IR oral solution (**d**), 25 mg CR tablet-A (**e**), and 200 mg CR tablet-C (**f**); and fed-state-200 mg CR tablet-D (**g**) and 200 mg CR tablet-E (**h**)-from different formulations.

The model was applied over a 5 mg IV infusion [18], internally verified using 10 mg IV infusion data [18], and externally validated with profiles of oral-dosage forms [13,23,24,25] while considering the fasted state (Figure 8).

For all oral-dosage forms used for the model verification (Figure 8), the predicted plasma concentration-time curves showed high R^2^ values: 0.994 (5 mg IV), 0.932 (10 mg IV), 0.879 (50 mg IR tablet), 0.997 (50 mg IR oral solution), 0.882 (25 mg ER tablet in fasted condition), 0.947 (95 mg ER tablet in fasted condition), 0.816 (200 mg ER tablet in fasted condition), 0.914 (200 mg ER tablet in fed condition), and 0.916 (200 mg ER tablet in fed condition). The predicted bioavailability from the formulations was about 40%, in line with the range of 38 ± 14% reported by Thummel et al. [43].

**Figure 9:**
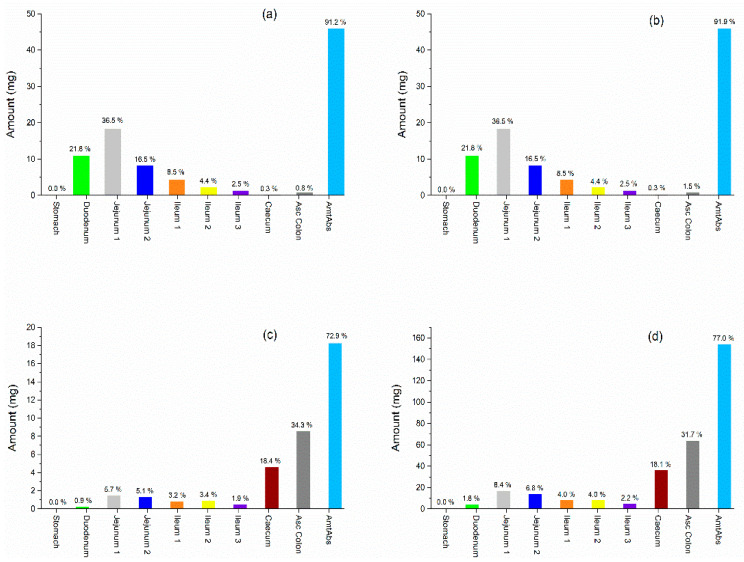
Prediction of regional absorption of metoprolol in gastrointestinal tract using different formulations: 50 mg IR tablet (**a**); 50 mg IR oral solution (**b**); 25 mg CR tablet-A (**c**); 200 mg CR tablet-C (**d**).

Our simulation’s results confirmed the predictions reported by Basu et al. [13], in which the IR formulations were primarily absorbed in the upper segments of the small intestine (duodenum and jejunum) due to their high solubility (BCS class I); while the ER formulations were absorbed in the lower segments (caecum and ascending colon) due to the controlled-release dosage form (Figure 9).

In vivo and in vitro data showed good matches based on the R^2^ values. The divergences between the observed and calculated points for the ER formulations can be justified by high variability in the metabolism, which may generate lower AUC values, as discussed by Lukacova et al. [22] and reported by Sirisuth and Eddington [44].

For each profile used to develop and validate the model, a statistical criterion was adopted: SC or %PE were calculated, which improved its robustness; these are summarized in Table 11 and Table 12, respectively.

**Figure 10:**
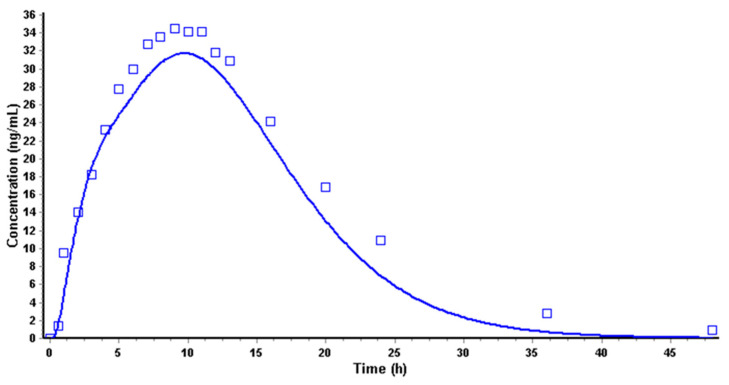
Observed (squares) and simulated (lines) plasma concentration-time curves of a 95 mg metoprolol succinate ER tablet, in which the simulation used the reference’s experimental dissolution profile that was obtained as described previously.

Moreover, the experimental dissolution profile of the 95 mg reference drug product (Selopress Zok^®^) was used to predict a plasma concentration-time curve that was compared with an in vivo curve reported by Ravishankar et al. [26]. There was a good match, and the calculated SC contemplated the simulated AUC_0−t_ and C_max_, as shown in Figure 10 and Table 13. Thus, the model was suitable for making predictions about the in vivo behavior of a formulation using its in vitro data as the input.

**Figure 11:**
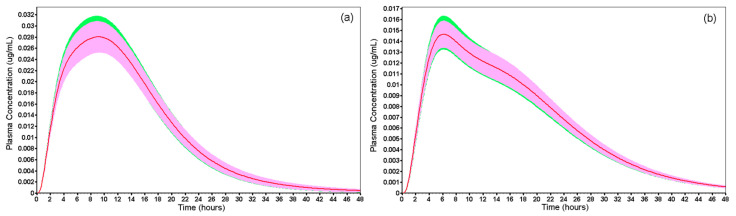
Virtual bioequivalence studies between the optimized formulation FO (green) and reference drug product Selopress Zok^®^ (pink), both containing 95 mg of metoprolol succinate, in fasted state (**a**) and fed state (**b**).

Finally, the verified model was used to perform the VBE studies. The reference drug product was compared with the developed mini-tablets. The optimized formulation FO, which showed the best in vitro performance, was subjected to VBE studies under fasted and fed states. The results of the VBE studies (Figure 11 and Table 13), showed that the FO was bioequivalent to Selopress Zok^®^ under both conditions.

Based on data obtained in the VBE, the 90% CI of the C_max_ and AUC_0–t_ for both the reference and FO were within the 80–125% interval (Table 13), and were in line with the regulatory guidance of the FDA [45] and ANVISA [46] for bioequivalence evaluation of test and reference drug products. In addition, the average AUC_(0–t)_/AUC_(0–inf)_ ratios for the test formulation (FO) and reference drug product in fasted and fed states were greater than 0.9, showing that the time length of 48 h set for the simulations was adequate (>80%) to efficiently predict the absorption.

## 4. Conclusions

It was possible to integrate DOE with PBBM to develop and optimize an extended-release mini-tablet formulation containing metoprolol succinate. The statistical design and evaluation of the formulations led to an understanding of the main parameters that controlled the drug release, thereby helping in the selection and optimization of the mini-tablet formulation. The obtained PK model and biopharmaceutical data analysis using PBBM helped to predict metoprolol succinate absorption for the mini-tablets. Thus, it was possible to use VBE, in conjunction with in vitro assays, to predict the performance of the optimized formulation, as well as to compare it to the reference one, in both fasted and fed states.

## Figures and Tables

**Table 1:** Composition of the MS mini-tablet formulations.

Components	F1	F2	F3
MS	40%	40%	40%
Microcrystalline cellulose 102	58%	18%	18%
Kollidon^®^ SR	-	40%	-
Methocel^®^ K100M	-	-	40%
Colloidal silicon dioxide	1%	1%	1%
Magnesium stearate	1%	1%	1%

**Table 2:** Assay matrix for coating mini-tablets—planning carried out using Statistica Program, version 11, 2012 (StatSoft Inc., Tulsa, OK, USA).

Assay	Core Formulation	Coating Weight Gain (%)	Kollicoat IR (%)
E1	F2	5	0
E2	F2	7.5	8
E3	F2	10	4
E4	F1	5	8
E5	F1	7.5	4
E6	F1	10	0
E7	F3	5	4
E8	F3	7.5	0
E9	F3	10	8

**Table 3:** Matrix of tests for mixtures of mini-tablets. Levels are shown depending on the number of mini-tablets used in the mixtures.

Test	E11	E12	F1
M1	5	10	1
M2	1	14	1
M3	3	12	1
M4	4	11	1
M5	2	13	1

**Table 4:** Summary of the key physicochemical and biopharmaceutical properties used to build the PK model of metoprolol in GastroPlus^®^.

Parameters	Value	Source
Molecular weight (g/mol)	267.37	ADMET Predictor^®^
LogD	−1.72 at pH 4.0	[19]
Solubility (mg/mL)	171 (pH = 6.5)	[20]
pKa	9.7 (Base)	[18]
Peff (cm/s × 10^−4^)	1.34 (Human)	[21]
Fup (%)	89	[22]
Blood-to-plasma ratio	1.13	[18]
Mean precipitation time (s)	900	Default setting

**Table 5:** Results of dissolution efficiency (DE%) obtained for MS from the coated mini-tablets and the reference drug product (Selopress Zok^®^).

Test	Core	Coating Weight Gain (WG%)	Kollicoat^®^ IR (%)	DE (%)
E1	F2	5	0	6.7
E2	F2	7.5	8	91.7
E3	F2	10	4	75.9
E4	F1	5	8	96.4
E5	F1	7.5	4	80.4
E6	F1	10	0	0.1
E7	F3	5	4	92.8
E8	F3	7.5	0	2.2
E9	F3	10	8	93.7
Reference	-	-	-	64.8

**Table 6:** Percentage of drug dissolved (Q) and dissolution efficiency (DE%) of the MS, obtained for the additional formulations and its reference, Selopress Zok^®^. The values for percentage of drug dissolved of the mini-tablets are expressed by the average of three determinations, and for the reference drug product by the average of 12 units.

Assay	IR *	Q%_1h_	Q%_4h_	Q%_8h_	Q%_20h_	DE (%)
E10	1%	0.8 ± 0.0	2.0 ± 1.5	4.6 ± 2.7	22.3 ± 3.8	11.0
E11	2%	0.9 ± 1.0	7.5 ± 2.3	28.9 ± 1.4	79.9 ± 4.8	47.3
E12	3%	0.5 ± 0.3	18.9 ± 1.3	56.3 ± 2.9	94.2 ± 2.7	63.3
Reference	-	9.4 ± 1.5	26.9 ± 1.4	51.6 ± 1.9	96.4 ± 3.5	64.8
USP specification [30]	-	Not less than 25%	Between 20–40%	Between 40–60%	Not less than 80%	-

* Kollicoat^®^ IR.

**Table 7:** Responses used to optimize the dissolution profile of MS. The values are expressed by the average of three determinations. In addition, the values of F1, E11, and E12 represent the number of mini-tablets used.

Assay	F1	E11	E12	Q%_2h_	Q%_4h_	Q%_10h_	Q%_20h_
M1	1	5	10	9.5 ± 0.2	20.1 ± 1.2	63.4 ± 1.7	71.8 ± 1.6
M2	1	1	14	9.8 ± 0.2	22.8 ± 1.3	70.5 ± 2.2	77.9 ± 2.0
M3	1	3	12	9.6 ± 0.4	21.5 ± 1.2	67.0 ± 2.0	74.8 ± 1.8
M4	1	4	11	9.6 ± 0.6	20.8 ± 1.2	65.2 ± 1.8	73.3 ± 1.7
M5	1	2	13	9.7 ± 0.3	22.1 ± 1.2	68.8 ± 2.1	76.4 ± 1.9

**Table 8:** Intended response sets in the optimization step based on the results of the reference product.

Parameter	Reference	Optimization
Q%_2h_	14.8 ± 0.8	Maximum
Q%_4h_	51.6 ± 1.9	Maximum
Q%_10h_	63.5 ± 2.2	Within the range
Q%_20h_	96.4 ± 3.5	Maximum

**Table 9:** Results of dissolution kinetics for the reference product and FO. The release constants (k_0_, k_H_, k_KP_), release coefficient (n), adjusted determination coefficients (R^2^_adj_.), Akaike criterion (AIC), and model selection criteria (MSC) were obtained for each model.

**Zero-Order**	**Reference**	**FO**
k_o_	0.08	0.08
R^2^_adj_.	0.9186	0.8861
AIC	86.93	91.16
MSC	2.34	2.00
**Higuchi**	**Reference**	**FO**
k_H_	2.56	2.57
R^2^_adj_.	0.9415	0.9223
AIC	82.96	86.48
MSC	2.67	2.40
**Korsmeyer–Peppas**	**Reference**	**FO**
k_KP_	0.35	0.54
n	0.80	0.73
R^2^_adj_.	0.9600	0.9787
AIC	79.25	82.30
MSC	2.98	2.74

**Table 10:** Summary of the two-compartment data for metoprolol calculated using the PKPlus™ module in GastroPlus^®^.

Parameters	Value
Clearance, CL (L/h)	76.67
Central compartment volume, Vc (L/Kg)	2.85
Elimination half-life, T_1/2_ (h)	3.05
Distribution rate constant from C1 to C2, K12 (h^−1^)	1.80
Distribution rate constant from C2 to C1, K21 (h^−1^)	2.50
Distribution volume of second compartment, V2 (L/Kg)	2.05

**Table 11:** In vivo and in silico data of IV and IR oral formulations used to develop and validate the model, as well as their respective %PE values.

Drug Record		IV 5 mg-10 min	IV 10 mg-5 min	IR-T 50 mg	OS 50 mg
C_max_ (µg/mL)	Observed	0.021	0.045	0.055	0.055
	Predicted	0.022	0.048	0.050	0.055
	%PE (%)	5.24	6.67	−9.09	0.01
AUC_0–t_ (µg·h/mL)	Observed	0.057	0.084	0.312	0.351
	Predicted	0.058	0.108	0.298	0.331
	%PE (%)	1.75	28.57	−4.49	−5.70

**Table 12:** In vivo and in silico data of ER oral formulations in fasted and fed states, adopted to develop and validate the model, as well as their respective SC ranges.

Drug Record		Fasted			Fed	
		A 25 mg	B 95 mg	C 200 mg	D 200 mg	E 200 mg
C_max_ (µg/mL)	Predicted	0.007	0.032	0.057	0.043	0.037
	LL—UL	0.005–0.011	0.023–0.046	0.041–0.069	0.039–0.057	0.031–0.044
AUC_0__−__t_ (µg·h/mL)	Predicted	0.119	0.529	1.111	0.889	0.925
	LL—UL	0.066–0.180	0.336–0.801	0.854–1.586	0.770–1.342	0.718–1.096

LL, lower limit of the SC; UL, upper limit of the SC.

**Table 13:** VBE data for 95 mg CR tablets in fasted and fed states.

Drug Record		Fasted		Fed	
		FO	Reference	FO	Reference
C_max_ (µg/mL)	Mean	0.031	0.030	0.016	0.015
	90% CI	0.027–0.033 87.31–105.73		0.014–0.017 89.60–107.99	
AUC_0–t_ (µg·h/mL)	Mean	0.518	0.510	0.311	0.313
	90% CI	0.461–0.560 88.86–108.11		0.288–0.338 92.60–108.88	
AUC_0–inf_ (µg·h/mL)	Mean	0.524	0.516	0.316	0.318
	90% CI	0.466–0.567 88.84–108.27		0.293–0.344 92.66–108.77	
Average AUC_0–t_/AUC_0–inf_		0.989	0.988	0.984	0.984

## Data Availability

The data presented in this study are available in the article and in the Supplementary Materials.

## Supplementary Materials

The following supporting information can be downloaded at: https://www.mdpi.com/article/10.3390/pharmaceutics14050892/s1, Table S1: Parameters used in the coating of the MS mini-tablets; Table S2: Composition (%) of coating films; Table S3: Characterization results of the MS mini-tablets. The values of average weight, true density, harness, and drug content are presented by the mean and standard deviation (SD) according to the number of determinations: 20, 5, 10, and 3, respectively; Table S4: Analysis of variance (ANOVA) for the dissolution efficiency (DE%) parameter. Results with a *p*-value < 0.05 were considered significant. Percentage of explained variation (R^2^_adjusted_) = 99.54; Table S5: Models used to analyze the results of the percentages of drug dissolved (Q%) at the times adopted as responses (2, 4, 10, and 20 h) and respective parameters derived from the analysis of variance and multiple regression to verify the adequacy of adjustment. *p*-values < 0.05 indicate the significance of the model; Table S6: Equations of the regression models used to optimize the MS dissolution profile for the mini-tablet mixtures.
