# Supplementary material for: Development of Extended-Release Mini-Tablets Containing Metoprolol Supported by Design of Experiments and Physiologically Based Biopharmaceutics Modeling
Source: Pharmaceutics. 2022 Apr 19;14(5):892. doi: 10.3390/pharmaceutics14050892 (PMC9146084; doi:10.3390/pharmaceutics14050892)
Supplement: Supplementary file 1 [file pharmaceutics-14-00892-s001.zip › pharmaceutics-1663967-supplementary.pdf]

pharmaceutics

MDPI

Supplementary Materials: Development of Extended-Release Mini-Tablets Containing Metoprolol Supported by Design of Experiments and Physiologically Based Biopharmaceutics Modeling

Michele Georges Issa, Natalia Vieira de Souza, Bruna Wenyi Chuang Jou, Marcelo Dutra Duque and Humberto Gomes Ferraz

Table S1. Parameters used in the coating of the MS mini-tablets.

Parameter	Preheat	Application	Drying
Inlet air temperature (°C)	50	50 to 70	50
Product temperature (°C)	42 to 45	42 to 55	40 to 45
Air flow ($m^3 h^{-1}$)	10	10 to 18	10 to 12
Time (min)	15	-	15
Coating nozzle diameter (mm)	-	0.8	-
Peristaltic pump speed (rpm)	-	3 to 4	-
Spray pressure (bar)	-	0.1 to 0.5	-
Microclimate pressure (bar)	-	0.05 to 0.1	-

Table S2. Composition (%) of coating films.

Components	E1	E2	E3	E4	E5	E6	E7	E8	E9	E10	E11	E12
Kollicoat® SR 30D	48.0	48.0	48.0	48.0	48.0	48.0	48.0	48.0	48.0	48.0	48.0	48.0
Kollicoat® IR (50:50 *w/w* mixture of White II:Brilliant Blue) [1]	-	13.7	6.9	13.7	6.9	-	6.9	-	13.7	1.7	3.4	5.1
Talc [2]	3.9	0.4	2.7	0.4	2.7	3.9	2.7	3.9	0.4	4.4	3.9	3.3
Titanium dioxide [3]	1.1	-	-	-	-	1.1	-	1.1	-	-	-	-
Purified water	47.0	37.9	42.4	37.9	42.4	47.0	42.4	47.0	37.9	45.9	44.7	43.6

[1] Corrected amount according to the percentage of Kollicoat® IR in the compositions of Kollicoat® IR White II and Kollicoat® Brilliant Blue = 58.1% (manufacture's information). [2] Added to complete 5% pigments in the coating composition (Kollicoat® IR White II and Kollicoat® Brilliant Blue contain 33.6% pigments (manufacture's information). [3] Used as an opacifying agent.

Table S3. Characterization results of the MS mini-tablets. The values of average weight, true density, hardness, and drug content are presented by the mean and standard deviation (SD) according to the number of determinations: twenty, five, ten, and three, respectively.

Formulation	Average Weight (mg) ± SD	True Density (g cm^{-3}) ± SD	Hardness (N) ± SD	Drug Content * ± SD	Friability (%)
F1	16.7 ± 0.6	1.43 ± 0.00	26.7 ± 4.4	40.2 ± 0.4	0.2
F2	16.4 ± 0.7	1.34 ± 0.01	36.4 ± 4.0	39.0 ± 0.3	0.5
F3	15.0 ± 0.7	1.37 ± 0.01	24.4 ± 4.3	40.3 ± 0.2	0.2

* Results of drug content expressed in grams of MS per 100 g of mini-tablets.

Table S4. Analysis of variance (ANOVA) for the dissolution efficiency (DE%) parameter. Results with p value < 0.05 are considered significant. Percentage of explained variation ($R^2_{adjusted}$) = 99.54.

Effect	Sum of Squares	Degrees of Freedom	Average of Squares	F	p
(1) Formulation	39.26	2	19.631	2.2631	0.306457
(2) Weight coating gain	130.46	2	65.231	7.5199	0.117372
(3) Kollicoat® IR	14793.02	2	7396.508	852.6780	0.001171
Error	17.35	2	8.674		

Table S5. Models used to analyze the results of the percentages of drug dissolved (Q%) at the times adopted as responses (2, 4, 10 and 20 h) and respective parameters derived from the analysis of variance and multiple regression to verify the adequacy of adjustment. p-value < 0.05 indicate the significance of the model.

Parameter	Model	p-value	R^2	$R^2_{adjusted}$
$Q\%_{2h}$	linear	0.0060	0.9423	0.9231
$Q\%_{4h}$	linear	<0.0001	0.9993	0.9991
$Q\%_{10h}$	linear	<0.0001	0.9999	0.9998
$Q\%_{20h}$	linear	<0.0001	0.9997	0.9997

Table S6. Equations of the regression models used to optimize the MS dissolution profile from the mini-tablet mixtures.

Response	Coefficients of the equations
$Q\%_{2h}$	0.58667 * E11 + 0.65667 * E12
$Q\%_{4h}$	0.89467 * E11 + 1.56467 * E12
$Q\%_{10h}$	3.04133 * E11 + 4.82133 * E12
$Q\%_{20h}$	5.23467 * E11 + 6.30467 * E12
